# Fracture of the Neck of the Os Femoris

**Published:** 1858-04

**Authors:** P. Spalding

**Affiliations:** Haverhill, N. H.


Communicated for the Boston Medical and Surgical Journal.

### *Fracture of the Neck of the os Femoris.*

Messrs. Editors.—In the *New England Journal of Medicine and Surgery*, No. 4, Vol. I., October, 1827, I reported a case of fracture of the cervix femoris within the capsular ligament, in which there was a perfect cure; and as an opportunity has since been obtained of an examination of the fracture, I have thought it might not be uninteresting, especially to the junior members of the profession, to republish in part the case, with the views then taken, together with the appearances on a *post-mortem* examination.

This patient was a man of feeble constitution, æt. 52, who on July 22d, fell in a saw mill, from a height of 16 feet, and struck on the left thigh and trochanter. When he was raised, the leg stood at an angle of about 40 degrees from the body, but it was immediately brought into place by an assistant. On examination a few hours after the accident, the foot and the knee were found everted, the limb was slightly shortened, but could easily be brought to its natural length, there was a sensible alteration in the position of the trochanter major, and when the patient was directed to bear on the affected limb, it gave him great pain in the region of the trochanter minor. The nature of the accident was made known, and constant extension for at least ninety days recommended. Objections were made by the patient to any appliances, more particularly on account of a pectoral affection, which rendered a recumbent position uncomfortable for any considerable length of time, and also in the hope that the accident might not be of so serious a character as represented.

The limb was placed in the most comfortable position, and directions were given the patient to remain as quiet as posible. On the 5th day it had contracted two and a half inches, and the eversion of the foot and knee was greater than on the day of the accident. By steady extension it could be brought to its natural length, but when this was romoved it would readily contract again with a slight jerk, and an increase of pain; this was greatly aggravated by rotation inward. There was but little swelling or external inflammation about the joint.

Sir Astley Cooper's views and treatment in cases of this character were examined, and made known to the patient, together with the reasons why they should not be adopted as a rule of practice. Mechanical appliances were instituted, and for seventy days constant extension as the circumstances indicated; after which, permission was given to sit up more or less, and move the limb with the greatest care without bearing any weight on it. In about four weeks after the limb was taken out of the apparatus, he began to walk with a cane, and shortly after recovered entirely, without any shortening or apparent eversion of the foot or knee.

For a description of the apparatus and its mode of application, reference is made to the full report of the case.

This patient died of phthisis pulmonalis nine years after the accident; and upon examination, the neck of the os femoris was found to have been fractured transversely near the head, and firmly united with ossific union, somewhat shortened and enlarged. A small spiculum of bone, about half an inch long, and one-fourth wide, lying near the cervix, seemed to grow out from that portion of the edge of the fracture belonging to the shaft. No considerations could induce friends to let me have this specimen for the benefit of science.

Whether this is the first well-attested case in this country of a perfect cure of fracture of the neck of the femur within the capsular ligament, I am not able to say; but it is believed that no one questioned the soundness of Sir Astley Cooper's views upon this subject until this case was reported. This eminent surgeon objected to making mechanical appliances in fracture of the neck of the os femoris within the capsular ligament—1st, on account of the difficulty of keeping up constant extension; 2d, of making lateral compression; 3d, that bony union never took place when the fracture was within the capsular ligament. In answer to these views, it was considerd that constant extension could be maintained, and had been, in the case above reported, and that lateral compression could be carried to any extent which the nature of the case required. Sir Astley's third objection, that bony union could not take place when the fracture was within the capsular ligament, because "the neck and head of the bone are supplied with blood from the periosteum of the cervix, and the reflected membrane which covers it, and that when the bone is fractured, if the periosteum be torn through, and the reflected membrane broken, to which there can be but very rare exceptions, all the means of ossific action are, in consequence of such fracture and laceration, necessarily destroyed in the head of the bone," required a more full consideration.

It was contended that the whole cavity of the joint was surrounded with the synovial membrane, which was reflected over the neck and head of the bone; that the bone is kept in place by strong ligaments; that the periosteum does not cover the head of the bone, or that portion within the capsular ligament; that the vessels which enter into the synovial membrane, and to form the round ligament, and nourish the head of the bone, are reflected from the acetabulum over the round ligament to the head of the bone, as well as from the neck, and that they inosculate with each other. In fracture of the cervix femoris, the reflected membrane may be broken, but the means of ossific action are not destroyed, as the head of the bone is nourished from vessels that pass from the acetabulum over the ligamentum teres, which keeps up its vitality. The same process which forms the bones in the fœtus, and promotes their growth and hardness, is carried on to unite them when fractured. This is retarded or facilitated according to circumstances. The situation of the fracture may render it slow, or old age and an impaired constitution may entirely suspend it, in parts endowed with a low degree of sensibility and vital action; but in a good constitution, when the broken extremities are reduced and kept in contact a sufficient length of time, ossific union will usually take place.

If parts destitute of periosteum cannot be united with bony union, as is contended, we are led to inquire by what action the extremities of several of the bones are formed, as of the femur, the olecranon process of the ulna, and the capsular extremity of the clavicle. Does not the same action produce the extremities of the bones that forms the other parts, differing only in degree, thereby requiring a longer time for the deposition of bony matter? And will it not as certainly unite them when they are fractured? If parts separated from the system, as the nose, ears and fingers, can be united, and do well; if the spur of the cock can be transplanted into the comb and thrive—can there be any inconsistency in believing that in fracture of the cervix femoris there remains sufficient vitality and natural action to repair the injury.

It was also observed that the experiments of Sir Astley Cooper were not conclusive, nor to be relied upon, because they were made upon animals, and could not be treated upon the correct principles of surgery; that it was impossible to produce a transverse fracture of the cervix femoris in an animal, and treat it so as to admit of ossific union, and therefore all such experiments were only calculated to mislead, and should not be relied upon in establishing principles of practice. From the anatomy and physiology of the hip-joint in the human subject, we have good reason to believe that ossific union would take place in fracture of the neck of the bone, when treated according to the nature of the accident and condition of the parts affected.

Since the above case was reported, many fine specimens of union of the neck of the femur have been collected, and the principle seems to be well established that ossific union may be expected in fracture of the neck of the femur within the capsular ligament, when all the indications are met.

It might be well to enquire why so few cases of this character are cured, and so many abandoned to die, or become cripples for life. Most fractures of the neck of the femur are in old people, or those with an impaired constitution, who are considered unable to bear the long confinement necessary to insure ossific union, and who would be better treated with reference to making them as comfortable as possible; but it is believed that many are left, who by judicious treatment might receive permanent cures. With our present improved appliances, every indication can be answered, and the long confinement necessary to a cure made quite easy.

In fractures in parts with a low degree of sensibility, or where ossific action does not readily take place, the treatment should be the more carefully attended to.

The sooner after the accident the bones are placed in opposition and maintained there, the greater probability of avoiding that inflammation in the cavity of the joint which produces a superabundance of synovia, which so often prevents the deposition of ossific matter.

It is highly probable that in every case, however favorable, there is an increased secretion of synovial fluid; but if lateral compression be perfectly maintained, so as to keep the fractured extremities in contact, absorption will take place, and bony matter be deposited in due time. In treating fractures of the neck of the femur within the cavity of the joint, sufficient attention is not paid to lateral compression. The limb may be brought down to its natural length in transverse fractures, and yet the ends of the bones may not coaptate; and, furthermore, if lateral compression is not sufficient to hold the broken extremities so firmly together that the acetabulum will roll on the head of the bone when the body is raised, leaving the fractured extremities perfectly at rest, then the head of the bone will be carried forward with the acetabulum, and break up all union that may have commenced.

As the broken surfaces are rough, they may be brought into perfect opposition and maintained, so that all necessary movements of the body may be made without endangering displacement. Whenever the body is to be moved in the least, the lateral compression should be correspondingly increased. Our appliances must be so perfectly arranged that every indication in the case may be fully answered, or we shall have no hope of effecting bony union.

In addition to mechanical appliances, and attention to the comfort of the patient, the general system should be carefully attended to. Some patients will require depletion and the antiphlogistic regimen; while others must be treated with tonics, stimulants and a generous diet.

If perfect extension be kept up for a few weeks, the contraction of the muscles will be so much overcome that the patient will suffer but little from it; but lateral compression should not be omitted at any time until permanent union has taken place. The least motion in the fractured extremities may break up provisional callus, and entirely destroy the recuperative power in the parts. Whenever lateral compression is abandoned, if union has not taken place, the inclination of the limb to rotation outward will most assuredly displace the bones, so as to prevent ossific union. We have seen cases which seemed to be of this character.

The difference between treating a case of fracture of the neck of the femur, and common fracture, is very great. While in the one case union readily takes place, and sometimes without surgical aid, in the other it is effected only by the most careful treatment.

Before undertaking to manage a case of this description, the surgeon should well consider whether he has skill, and appliances to fully meet every indication, or he should attempt nothing. It is better to "own up at once," than to fail for the want of those means without which we cannot expect success.

P. SPALDING, M. D.

*Haverhill, N. H., Feb. 4th,* 1858.

